# Early outcome and blood-brain barrier integrity after co-administered thrombolysis and hyperbaric oxygenation in experimental stroke

**DOI:** 10.1186/2040-7378-3-5

**Published:** 2011-06-16

**Authors:** Dominik Michalski, Johann Pelz, Christopher Weise, Johannes Kacza, Johannes Boltze, Jens Grosche, Manja Kamprad, Dietmar Schneider, Carsten Hobohm, Wolfgang Härtig

**Affiliations:** 1 Department of Neurology, University of Leipzig, Liebigstr. 20, 04103 Leipzig, Germany; 2 Paul Flechsig Institute for Brain Research, University of Leipzig, Jahnallee 59, 04109 Leipzig, Germany; 3 Department of Anatomy, Histology and Embryology, Faculty of Veterinary Medicine, University of Leipzig, An den Tierkliniken 43, 04103 Leipzig, Germany; 4 Fraunhofer Institute for Cell Therapy and Immunology, Perlickstr. 1, 04103 Leipzig, Germany; 5 Translational Centre for Regenerative Medicine, University of Leipzig, Philipp-Rosenthal-Str. 55, 04103 Leipzig, Germany; 6 Institute of Clinical Immunology and Transfusion Medicine, University of Leipzig, Johannisallee 30, 04103 Leipzig, Germany

## Abstract

**Background:**

After promising results in experimental stroke, normobaric (NBO) or hyperbaric oxygenation (HBO) have recently been discussed as co-medication with tissue plasminogen activator (tPA) for improving outcome. This study assessed the interactions of hyperoxia and tPA, focusing on survival, early functional outcome and blood-brain barrier (BBB) integrity following experimental stroke.

**Methods:**

Rats (n = 109) underwent embolic middle cerebral artery occlusion or sham surgery. Animals were assigned to: Control, NBO (60-minute pure oxygen), HBO (60-minute pure oxygen at 2.4 absolute atmospheres), tPA, or HBO+tPA. Functional impairment was assessed at 4 and 24 hours using Menzies score, followed by intravenous application of FITC-albumin as a BBB permeability marker, which was allowed to circulate for 1 hour. Further, blood sampling was performed at 5 and 25 hours for MMP-2, MMP-9, TIMP-1 and TIMP-2 concentration.

**Results:**

Mortality rates did not differ significantly between groups, whereas functional improvement was found for NBO, tPA and HBO+tPA. NBO and HBO tended to stabilize BBB and to reduce MMP-2. tPA tended to increase BBB permeability with corresponding MMP and TIMP elevation. Co-administered HBO failed to attenuate these early deleterious effects, independent of functional improvement.

**Conclusions:**

The long-term consequences of simultaneously applied tPA and both NBO and HBO need to be addressed by further studies to identify therapeutic potencies in acute stroke, and to avoid unfavorable courses following combined treatment.

## Background

The repeated translational failures of preclinical approaches in acute ischemic stroke necessitate a more complex perspective of tissue salvaging and regeneration in experimental research [1], involving functional key-structures such as the blood-brain barrier (BBB) with the associated 'neurovascular unit' [2-5]. Thromboembolic models are considered to provide best comparability to the human pathophysiology, thus being of increasing interest [5,6]. Along with the growing knowledge on time-dependent BBB changes [7,8], matrix metalloproteinases (MMPs) and their inhibitors (TIMPs) were proposed as mediators with deleterious effects in early phases, but also regenerative properties in subsequent phases [9,10]. The application of oxygen under normobaric (NBO) or hyperbaric (HBO) conditions was shown to be beneficial in experimental studies by decreasing infarct size, functional impairment, hemorrhagic transformation, and early MMP response leading to BBB stabilization [11-17]. Recently, hyperoxia was discussed as co-medication to tissue plasminogen activator (tPA) [17,18], which even results in clinical improvement [19], but is also known for MMP activation and BBB breakdown with possible hemorrhagic transformation [20-22]. Hence, hyperoxia might attenuate deleterious tPA effects and improve the clinical outcome [18]. Our group previously found that simultaneous treatment with tPA and HBO may have detrimental effects on long-term outcome in rats, possibly by influencing BBB integrity due to early interactions [23]. The present study aimed on interacting effects of hyperoxia and tPA with special emphasis on survival, early functional outcome, BBB integrity and related mediators in a translational relevant setting after thromboembolic stroke in rats.

## Methods

### Experimental design and interventions

The experimental protocol involving animals was approved by local authorities [Regierungspräsidium Leipzig, Tierversuchsvorhaben (TVV) 02/09] and has been conducted according to the European Communities Council Directive (86/609/EEC). Male Wistar rats (mean weight 316.2 g), provided by Charles River (Sulzfeld, Germany), underwent right-sided middle cerebral artery occlusion (MCAO; n = 105) or sham operation (n = 4), as described below. Thirteen animals died during surgery and 4 animals were excluded from the study due to minor functional impairment (Menzies score 0-1) indicating insufficient cerebral ischemia, as specified previously. Finally, 92 animals were consecutively assigned to sham surgery (n = 4), MCAO control (n = 17), NBO (n = 18), HBO (n = 16), tPA (n = 19), or HBO+tPA (n = 18). Treatment was initiated 2 hours after ischemia onset: The NBO group received 60 minutes of normobaric pure oxygen in a hyperbaric chamber (Sayers/Hebold, Cuxhaven, Germany), the HBO group underwent 60 minutes (plus compression/decompression) of 100% O_2 _at 2.4 absolute atmospheres (ATA). tPA-treated animals received 9 mg/kg bodyweight tPA (Actilyse, Boehringer, Ingelheim, Germany) intravenously over 30 minutes. The HBO+tPA group simultaneously received tPA and HBO beginning at 2 hours. Functional impairment was assessed after surgery (baseline) and 4- or rather 24-hour observation period post MCAO, iimmediately followed by fluorescein isothiocyanate (FITC)-albumin injection *via *a femoral vein (20 mg/1 mL saline; Sigma, Taufkirchen, Germany) for visualization of BBB permeability. After usually 1 hour of circulation, animals were sacrificed in deep narcosis. Blood samples were drawn transcardially, followed by perfusion with saline and 4% paraformaldehyde in phosphate-buffered saline (PFA). Brains were removed from skull, immersed in the same fixative for 24 hours and equilibrated in 30% buffered sucrose. All study endpoints were defined prior to the first animal enrollment.

### Surgical procedure

MCAO was induced using an embolic model according to Zhang et al. [24] with minor modifications as described previously [23]. Briefly, a polyethylene (PE) tubing with tapered end was advanced from an incision of the external carotid artery (ECA) into the internal carotid artery, reaching the origin of the middle cerebral artery. A weight-adapted blood clot (mean length 46 mm) was injected, followed by catheter removal and ligation of the ECA stump. The sham operation was performed in the same manner, including preparation of cervical vessels, but without catheter insertion. In all groups, PE tubes were inserted into the femoral vein for tPA administration and the femoral artery for monitoring heart rate, arterial pressure (Datex, Helsinki, Finland), and obtaining blood samples (PaO_2 _and glucose; ABL 700, Radiometer, Copenhagen, Denmark) during surgery and after therapy. Animals were anesthetized using 2-2.5% isoflurane (70% N_2_O/30% O_2_) during surgery. Thereby, the body temperature was adjusted to 37.0°C using a thermostatically controlled heating pad (Fine Science Tools, Heidelberg, Germany) with rectal probe.

### Assessment on mortality and functional impairment

The rate of premature death was recorded in each treatment group during the observation period including associated FITC-albumin injection. The Menzies score was used to assess functional impairment [25], scaled from 0 (no apparent deficits) up to 4 (spontaneous contralateral circling).

### Tissue preparation, imaging and quantification of BBB permeability

**Figure 1:**
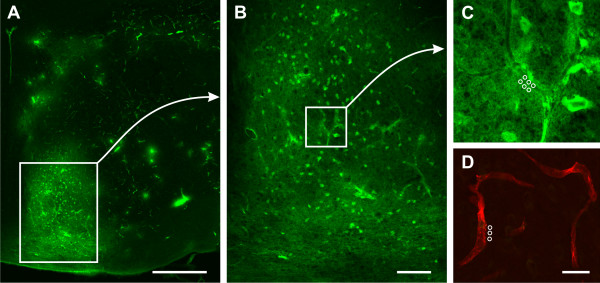
**Demonstration of enhanced FITC-albumin measurement as BBB leakage marker at 5 and 25 hours after embolic stroke in rats by confocal laser scanning micrographs**. (A), representative striatal ischemia-related leakage of Cy2-enhanced FITC-albumin at 5× magnification in a NBO-treated animal at 5 hours. (B), enhanced FITC-albumin in ischemic border zone at 10× magnification showing impaired vascular structures with leakage phenomena. (C), para- and intravasal inserted region of interests at 63× magnification. (D), 63× magnification of the corresponding area at the non-ischemic hemisphere: Detection of FITC-albumin background staining combined with Cy3-immunolabeling of SMI-71 for vessel localization. Absent leakage indicates BBB integrity. Scale bars: in A = 500 μm, in B = 100 μm, and in D = 25 μm.

The visualization technique of BBB impairment after *in vivo *application of FITC-albumin as permeability marker was described previously [26]. Briefly, 30 μm-thick serial brain sections were cut coronally using a freezing microtome. Ten series of sections per animal were stored in 0.1 mol/L Tris-buffered saline (TBS; pH 7.4) containing sodium azide. After washing, sections were mounted on fluorescence-free slides, air-dried and coverslipped with Entellan in toluene (Merck, Darmstadt, Germany). Subsequent to a screening microscopy (Axioplan, Zeiss, Jena, Germany) for identification of sections with the clearest infarct presentation by FITC-albumin leakage, the neighboring serial sections were blocked (TBS, 2% bovine serum albumin, 0.3% Triton X-100 [TBS-BSA-T]), followed by an enhancement of the green fluorescent signal with carbocyanine (Cy)2-anti-FITC IgG (20 μg/mL TBS-BSA-T; Jackson ImmunoResearch, West Grove, PA) for long-term stabilization. Moreover, vascular membranes were detected by SMI-71 (1:400; Emeryville, CA [27]) and Cy3-donkey-anti-mouse IgM (20 μg/mL TBS-BSA; Jackson), colored in red. Finally, washing, mounting and coverslipping were performed as described above. Fluorescence-based paravasal intensity of enhanced FITC-albumin served as surrogate for BBB permeability, as exemplarily shown in Figure 1, and was analyzed by a blinded investigator. For this purpose, a fluorescence microscope (Axioplan 2, Zeiss) with structured-light confocal system (OptiGrid, Qioptiq, Fairport, NY), digital camera (ORCA-ER, Hamamatsu, Hamamatsu City, Japan) and imaging software (Volocity 4.3, PerkinElmer, Waltham, MA) was used. Routinely, 3 vessels in the lesion-bordering zone with clearest leakage phenomena were selected. Six measurements were performed for each of the chosen vessels by using cylindrical region of interests (ROIs; diameter 2 μm) with 9 confocal layers (z-interval 0.5 μm) at each point, obtained at 40x magnification using a water immersion objective (numerical aperture 1.4), with further digital zoom if necessary. ROIs were positioned intravasally (n = 3) and with distance of approximately 2 μm from the vessel wall (n = 3). To control for any background staining, 1 corresponding vessel in the contra-lesional hemisphere was identified by SMI-71/Cy3 and paravasal space was measured by 3 ROIs in the same manner. For all measurements the following setup was applied: Exposure time 80 ms, gain 0, offset 0, super grid quality, grid gain 3x, excitation Cy2 480 nm, emission 527 nm, excitation Cy3 545 nm, emission 610 nm. The obtained mean intensity within the ROIs was used for further processing. Finally, means of 3 ipsi-lesional vessels and 1 contra-lesional vessel were calculated and subtracted for total leakage. Furthermore, a surrogate for BBB integrity was calculated as extra-/intravasal ratio on the affected hemisphere indicating the leakage proportion. For optimal presentation, micrographs of fluorescence labeling were obtained using a 510 Meta laser scanning microscope (Zeiss), equipped with an argon laser (488 nm) for the excitation of Cy2, and two helium-neon lasers for the excitation of Cy 3 (543 nm). For the detection of emitted spectra, the following band-pass filters were used: 500-530 nm for Cy2 (green), and 565-615 nm for Cy3 (red). The original images were processed with Adobe Photoshop 7.0 (Adobe Systems Inc., Mountain View, CA). Thereby, brightness, contrast and sharpness of the final pictures were slightly modified.

### Measuring BBB-related serum markers

Samples were allowed to clot, and then centrifuged and finally stored up to -80°C. After thawing, MMP-2, MMP-9, TIMP-1 and TIMP-2 concentrations were determined in a blinded fashion by using enzyme-linked immunosorbent assays (USCN, Wuhan, China; E90100Ra/E90553Ra/E90552Ra/E90128Ra). The minimum detectable dose, which was differentiable from zero (as listed in the instruction manual), was utilized for values under the minimum or with uncertain calculation at the lower standard curve.

### Statistical analysis

All calculations were performed with SPSS Vers. 18.0 (SPSS Inc, an IBM Company, Chicago, IL). One-way analysis of variance (ANOVA) was applied for inter-group differences at a single time point, followed by the Duncan test considering multiple means. For intra-group testing at different time points, Wilcoxon-Mann-Whitney and Kolmogorov-Smirnov tests were performed. Percentage rates were verified using the Chi-square test with additional Monte Carlo simulation (confidence interval = 99%, samples = 10,000), ensuring exact testing. Pearson correlations were applied for interrelations. A *P *< 0.05 was considered as statistically significant.

## Results

Physiological parameters were similar between groups with exception of weight (slightly increased in sham- and tPA-treated animals), and marginal differences in temperature, mean arterial pressure and PaO_2 _- overall indicating no major group differences (Table 1). As expected for this model, ischemia predominantly occurred in the proximal section of the middle cerebral artery territory with varying infarct sizes as indicated by FITC-albumin leakage.

**Table 1:** Physiological parameters.

	Sham	Control	NBO	HBO	tPA	HBO+tPA	ANOVA
	mean ± SD	mean ± SD	mean ± SD	mean ± SD	mean ± SD	mean ± SD	F (*P*)
							
*Weight (g)*	357.8 ± 10.9	324.1 ± 45.6	304.3 ± 26.2	296.9 ± 26.5	334.8 ± 36.6	317.0 ± 17.5	4.6 (**)†
*Duration of surgery (minutes)*	63.8 ± 9.1	74.5 ± 25.5	66.6 ± 18.2	71.0 ± 22.8	68.3 ± 14.7	58.1 ± 12.6	1.5 (n.s.)
							
*Temperature (°C)*							
Initial	37.5 ± 0.4	37.5 ± 0.4	37.6 ± 0.5	37.7 ± 0.3	37.5 ± 0.3	37.6 ± 0.5	0.7 (n.s.)
After catheter	37.6 ± 0.2	37.3 ± 0.3	37.2 ± 0.3	37.1 ± 0.4	37.2 ± 0.7	37.2 ± 0.4	0.9 (n.s.)
After clot injection	37.5 ± 0.0	37.4 ± 0.2	37.3 ± 0.3	37.2 ± 0.3	37.3 ± 0.7	37.1 ± 0.6	1.1 (n.s.)
End of surgery	37.6 ± 0.4	37.5 ± 0.3	37.2 ± 0.2	37.3 ± 0.3	37.4 ± 0.4	37.0 ± 0.5	3.6 (**)‡
After therapy	n/a	n/a	36.8 ± 0.8	37.3 ± 0.7	37.2 ± 0.8	36.9 ± 0.8	1.7 (n.s.)
							
*Mean arterial pressure (mmHg)*							
After catheter	75.7 ± 3.5	82.9 ± 6.5	78.1 ± 8.4	77.8 ± 8.4	74.4 ± 9.0	79.9 ± 8.2	2.2 (n.s.)
After clot injection	72.5 ± 3.5	78.2 ± 6.3	73.3 ± 5.8	77.1 ± 9.0	72.4 ± 7.9	77.7 ± 11.8	1.5 (n.s.)
End of surgery	62.0 ± 3.0	75.3 ± 8.3	74.8 ± 13.2	79.1 ± 11.7	71.4 ± 7.2	81.9 ± 12.6	3.0 (*)§
After therapy	n/a	n/a	96.0 ± 15.5	86.9 ± 10.7	84.4 ± 12.9	86.9 ± 17.0	2.1 (n.s.)
							
*Heart rate (per minute)*							
After catheter	206.7 ± 42.5	188.5 ± 17.5	189.3 ± 12.6	191.5 ± 21.8	197.0 ± 18.5	192.7 ± 26.9	0.7 (n.s.)
After clot injection	220.0 ± 49.5	194.5 ± 12.0	187.9 ± 17.0	203.1 ± 27.7	192.4 ± 21.7	203.9 ± 23.2	1.8 (n.s.)
End of surgery	220.0 ± 40.9	198.5 ± 12.3	200.6 ± 25.3	200.9 ± 22.2	192.8 ± 18.9	200.2 ± 19.0	1.0 (n.s.)
After therapy	n/a	n/a	203.1 ± 13.3	194.5 ± 13.0	204.2 ± 18.0	212.2 ± 21.7	2.7 (n.s.)
							
*PaO*_*2 *_*(mmHg)*							
After catheter	97.4 ± 9.1	102.8 ± 13.7	104.2 ± 22.6	97.9 ± 14.5	98.6 ± 9.2	105.1 ± 12.9	0.7 (n.s.)
End of surgery	105.3 ± 12.3	114.1 ± 29.5	118.6 ± 19.9	108.5 ± 18.3	107.6 ± 13.8	114.7 ± 14.5	0.8 (n.s.)
After therapy	n/a	n/a	117.8 ± 23.9	118.4 ± 18.2	117.7 ± 13.6	149.8 ± 28.9	8.8 (***)II
							
*Blood glucose (mmol/l)*							
After catheter	13.5 ± 1.7	10.5 ± 1.9	12.2 ± 2.3	11.8 ± 1.5	11.1 ± 2.5	11.4 ± 1.6	2.1 (n.s.)
End of surgery	13.2 ± 1.5	10.4 ± 1.9	11.8 ± 3.3	10.6 ± 1.6	11.2 ± 3.1	11.6 ± 2.4	0.9 (n.s.)
After therapy	n/a	n/a	9.2 ± 1.8	10.0 ± 1.8	8.8 ± 1.4	9.9 ± 1.5	2.2 (n.s.)
							

*P*: *<0.05, **<0.01, ***<0.001; Duncan test: †, sham/tPA > control/NBO/HBO/HBO+tPA; ‡, HBO+tPA < sham/control/HBO/tPA; §, sham < control/NBO/HBO/tPA/HBO+tPA; II, HBO+tPA > NBO/HBO/tPA

### Mortality and early functional impairment

**Figure 2:**
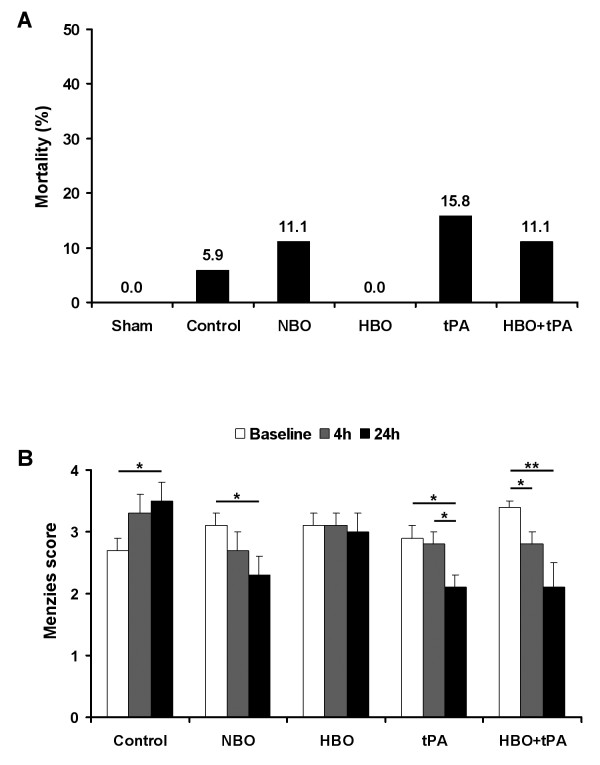
**Mortality rates (A) during observational period depending on intervention**. Sample size (sham/control/NBO/HBO/tPA/HBO+tPA): 4/17/18/16/19/18. Time course of functional impairment (B) depending on intervention. Sample size at baseline (control/NBO/HBO/tPA/HBO+tPA): 17/18/16/19/18, at 4 hours: 11/11/16/18/16, at 24 hours: 9/8/8/8/8. Bars: means, lines: standard errors, *P*-value: *<0.05, **<0.01.

Premature death occurred in a total of 8 animals (8.7%). The distribution according to the treatment groups is shown in Figure 2A, and was not statistically significant (Chi-square, *P *= 0.617; Monte Carlo, *P *= 0.678). As expected, no functional impairment was found in sham-assigned animals. Figure 2B indicates the course of functional impairment depending on treatment. At baseline and 4 hours, impairment did not differ between the groups (ANOVA, *P *= 0.073, *P *= 0.437); at 24 hours differences were found (ANOVA, *P *= 0.004) with superiority for NBO, tPA and HBO+tPA compared with control and HBO (Duncan, *P *< 0.05). Regarding the time course, controls showed a progressive functional impairment during the first 24 hours (Wilcoxon-Mann-Whitney, *P *= 0.044), whereas improvement was noted for NBO, tPA and HBO+tPA (Wilcoxon-Mann-Whitney, NBO-baseline/24 hours *P *= 0.044, tPA-baseline/24 hours *P *= 0.023, 4/24 hours *P *= 0.039, HBO+tPA-baseline/4 hours *P *= 0.006, baseline/24 hours *P *= 0.048). No time-dependent changes appeared in HBO-treated animals.

### BBB permeability

**Figure 3:**
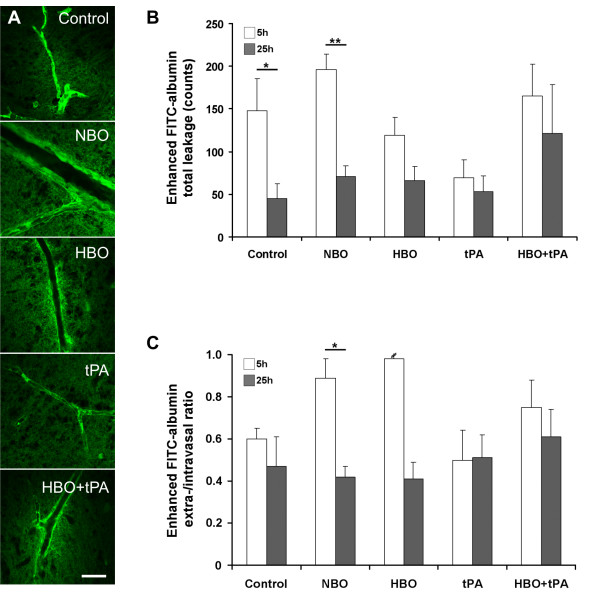
**Treatment-dependent representative samples of impaired vessels (A) at 5 hours displayed by confocal laser scanning micrographs (100× magnification; scale bar = 20 μm)**. Total paravasal leakage (B) of FITC-albumin on ischemic hemisphere considering background staining as detected on contra-lesional hemisphere and extra-/intravasal ratio (C), calculated by means on affected hemisphere. Sample size for leakage and ratio at 5 hours (control/NBO/HBO/tPA/HBO+tPA): 8/8/8/7/8, at 25 hours: 8/8/8/8/8. Bars: means, lines: standard errors, *P*-value: *<0.05, **<0.01.

FITC-albumin leakage was not observed in sham animals (data not shown). In controls, total leakage declined from 5 to 25 hours (Figure 3A). Concerning the treatment, total leakage differed at 5 hours (ANOVA, *P *= 0.046) between tPA and NBO, as well as between tPA and HBO+tPA (Duncan, *P *< 0.05 each). HBO tended to reduce leakage, but this was not statistically significant. At 25 hours, no group-specific differences of total leakage occurred (ANOVA, *P *= 0.413), whereas combined treatment with HBO and tPA tended to increased leakage. Concerning the time course, controls and NBO-treated animals showed a significant reduction from initial values, indicating dynamic properties of BBB integrity (Kolmogorov-Smirnov, *P *= 0.022, *P *= 0.004).

Extra-/intravasal ratios (Figure 3B) did not differ between treatment groups at either 5 or 25 hours (ANOVA, *P *= 0.097, *P *= 0.682). At 25 hours, oxygen application (NBO or HBO) tended to have the lowest leakage proportions, in contrast to worsened tPA- and HBO+tPA-treated animals. Time-dependent changes were solely found for NBO with a decreasing value (Kolmogorov-Smirnov, *P *= 0.022). The leakage proportion of the whole sample significantly correlated with functional impairment at baseline and 4 hours (Pearson, *r *= 0.391, *P *= 0.000, *r *= 0.392, *P *= 0.001), but failed to correlate at 24 hours (Pearson, *r *= 0.186, *P *= 0.251).

### BBB-related serum markers

**Figure 4:**
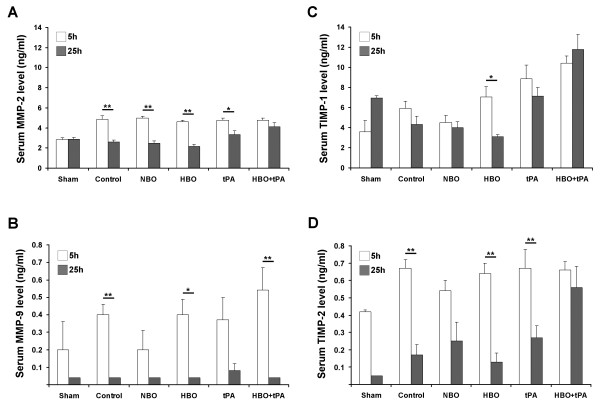
**Serum levels of MMP-2 (A), MMP-9 (B), TIMP-1 (C), and TIMP-2 (D), obtained during final narcosis at 5 or 25 hours**. Sample sizes for MMP-2, MMP-9, TIMP-1, and TIMP-2 at 5 hours (sham/control/NBO/HBO/tPA/HBO+tPA): 2/8/8/8/7/8, at 25 hours: 2/8/8/7/8/8. Bars: means, lines: standard errors, *P*-value: *<0.05, **<0.01.

As shown in Figure 4, significantly lower MMP-2 levels were detected in sham animals at 5 hours as compared to all other groups (ANOVA, *P *= 0.012; Duncan, *P *< 0.05). At 25 hours, HBO resulted in decreased MMP-2 levels as compared to tPA and HBO+tPA (ANOVA, *P *= 0.001; Duncan, *P *< 0.05). Concerning the time course, MMP-2 levels decreased in controls, and NBO-, HBO- and tPA-treated animals (Kolmogorov-Smirnov, *P *= 0.001, *P *= 0.001, *P *= 0.001, *P *= 0.037). As shown in Figure 4B, no significant treatment-related differences occurred for MMP-9 (ANOVA, 5 hours *P *= 0.316, 25 hours *P *= 0.554), but decreasing levels between 5 and 25 hours were found in controls, HBO- and HBO+tPA-treated animals (Kolmogorov-Smirnov, *P *= 0.004, *P *= 0.030, *P *= 0.004). For TIMP-1 (Figure 4C), tPA and HBO+tPA-assigned animals revealed increased levels at 5 hours compared to sham, control and NBO (ANOVA, *P *= 0.000; Duncan, *P <*0.05). At 25 hours, HBO+tPA showed the highest TIMP-1 levels, followed by tPA/sham and HBO (ANOVA, *P *= 0.000; Duncan, *P *< 0.05). For HBO, the TIMP-1 levels significantly declined between 5 and 25 hours (Kolmogorov-Smirnov, *P *= 0.037). TIMP-2 (Figure 4D) demonstrated no treatment-specific differences at 5 hours (ANOVA, *P *= 0.388), but the co-administration of HBO and tPA led to increased TIMP-2 levels at 25 hours as compared to HBO, control and sham (ANOVA, *P *= 0.011; Duncan, *P *< 0.05). A TIMP-2 reduction between 5 and 25 hours was noted for controls, HBO- and tPA-treated animals (Kolmogorov-Smirnov, *P *= 0.001, *P *= 0.001, *P *= 0.007).

## Discussion

For the first time, the present data provide insights into complex BBB processes following hyperoxia, supplemented by testing HBO and tPA simultaneously. This study focused on changes of BBB integrity in the early stages following stroke due to their potential long-term relevance. In consideration of translational aspects [5,6], an embolic model was chosen and treatment initiated after ischemia onset without preconditioning attempts.

The mortality rates did not differ significantly between groups, notably for the combined treatment with HBO and tPA. This finding reinforced previous reports that focused on additional NBO to tPA and provided comparable results when applied simultaneously [28], or consecutively by starting with NBO [12,29]. Functional impairment deteriorated in controls during first 24 hours emphasizing progressive stroke, contrary to significant improvement following NBO, tPA and HBO+tPA treatment. Previous reports on the short-term beneficial effects of tPA [23,28] are confirmed by the current data. The surprising lack of HBO efficacy raises several questions such as those of the pressure used (2.4 ATA), possibly suboptimal to obtain beneficial effects, but chosen due to well tolerability in patients with other diseases (e.g., problem wounds). However, previous data on hyperoxia in experimental stroke indicate efficacy predominantly in transient *versus *permanent ischemia [13,17].

By investigating leakage phenomena on vascular structures at 2 different time points, mechanisms of ischemia-induced BBB damage were addressed quantitatively. Previous studies investigated extravasation mainly by quantifying Evans Blue [7,30,31], sodium fluorescein [16] or FITC-dextran [32] directly, or even by gadolinium-enhanced magnetic resonance imaging [8,31,33]. FITC-albumin has advantages as inert vehicle [26] and is comparable with FITC-dextran regarding molecular structure, which revealed significant associations to infarct size and impaired neuronal structures as shown by Fluoro-Jade staining [32]. Concerning the time course of BBB impairment basically 2 hypotheses exist: Long-lasting BBB opening (e.g., several weeks) has been reported shortly after ischemia/reperfusion [31,33], whereas other authors reported a biphasic dynamics [7,8]. As shown in controls, total leakage decreased from 5 to 25 hours, whereas the leakage proportion did not differ between 5 and 25 hours, indicating early maximal BBB alteration. However, the comparison is limited by different types of ischemia, more precisely, thromboembolic in the present study *versus *filament-induced ischemia/reperfusion in previous reports [7,8,31,33]. Concerning the treatment, the present data revealed the lowest total leakage for tPA at 5 hours, most likely related to early recanalization, and tended to an improved leakage proportion for hyperoxia at 25 hours indicating BBB stabilization. As expected, use of tPA worsened BBB integrity at 25 hours, while co-administered HBO showed no attenuation of this effect. The loss of integrity is mainly attributed to MMP activation due to enzymatic effects of tPA [21,34], as previously shown by a tPA dose-dependent correlation of BBB leakage and MMP-9 levels [20]. Interestingly, the leakage proportion solely correlated with early functional impairment, indicating that worsened BBB integrity following tPA and HBO+tPA were not clinically relevant at 24 hours.

MMPs as proteolytic enzymes known to degrade extracellular matrix and corresponding TIMPs with primarily inhibiting functions [9,21], were measured to reveal mechanisms of BBB regulation. Park et al. [35] demonstrated a significant correlation between MMP-9 values in blood and brain tissue, which enables parallel serum investigation and brain tissue analysis by immunohistochemistry. Previous studies associated vascular disruption with MMP-2 [36] and MMP-9 [30,37]. This becomes relevant in tPA-related hemorrhagic transformation [22,38], because tPA raises MMPs and TIMPs as consequence of plasminogen activation [21,34,39]. Prior studies revealed an inconsistent time course of MMPs, whereas here MMP-2 levels were found to increase within hours after ischemia mediating potentially initial BBB opening, which might be followed by a MMP-9-related second opening during the first days [9,38,40]. Other authors noted that MMP-9 responses in the acute phase, and afterwards MMP-2 in later phases after stroke [10]. These conflicting data may result from different measurement techniques, ischemia models (transient/permanent) and animal species. Here, the MMP-9 levels at most tended to increase at 5 hours but markedly decreased at 25 hours when compared to sham, coherent with reports on later peak elevation [9,40]. Overall, HBO led to the lowest MMP-2 levels at 25 hours (closest to NBO) and decreased MMP-9 during the first 25 hours, which is in accordance with low leakage. Veltkamp et al. [15] also demonstrated a MMP-9 reduction with corresponding decreased basal lamina degradation at 24 hours following HBO in experimental stroke. tPA resulted in an elevated MMP-2 level, but unexpectedly HBO failed to attenuate this effect with concomitant use. The data on TIMPs resembled those of the MMPs: At 25 hours, HBO led to the lowest values and tPA-associated interventions resulted in increased TIMPs. Currently, causal links between MMPs and TIMPs remain unresolved due to different interpretations: TIMPs might be reactively elevated corresponding with MMPs, or following stroke itself even before MMPs increased, further complicated by known paradoxic (exactly not only inhibiting) roles of TIMPs [21,41]. Future stroke studies on MMP modification should consider the complexity of MMPs acute deleterious effects and their known delayed beneficial actions in recovery [9,10]. Zhao et al. [42] impressively demonstrated decreased infarct sizes by inhibition of MMP at day 1, but infarct worsening by inhibition at day 7.

The present study has some limitations: Intracerebral hemorrhage or recanalization rates were not determined, but complications were considered in mortality and functional impairment. The 24-hour observation period limits generalization and implies further research on BBB integrity during longer periods. Due to the existence of previous reports regarding NBO simultaneously applied to tPA [28], this combination has not been investigated, although it would be easily to implement into clinical routine. HBO was performed only at 2.4 ATA; a higher pressure would possibly stabilize BBB integrity, even when co-administered with tPA.

## Conclusions

In summary, NBO showed promising results with decreased functional impairment and reduced BBB permeability during the first 25 hours after acute experimental stroke. Surprisingly, HBO failed to demonstrate functional improvement, but tended to stabilize BBB and to reduce MMP activation. Concomitant treatment with HBO and tPA provided early functional improvement, potentially due to enhanced recanalization, but caused increased BBB permeability and MMP-2 activation, which could impede delayed regenerative processes. Further studies are required to identify long-term interactions of tPA with both NBO and HBO, especially in order to avoid unfavorable courses of combined treatment. Additionally, different duration and altered pressure of oxygen application together with tPA need to be addressed in future studies to explore potential time- and pressure-related side effects.

## Competing interests

The authors declare that they have no competing interests.

## Authors' contributions

DM, JB, CH, JK and WH conceived the present study including outcome measurements. DS facilitated funding. DM and CW conducted the animal experiments. JP and WH performed tissue preparation and serial tissue staining. JG carried out laser scanning microscopy. DM and CW recorded the functional outcome; further endpoints were assessed in a blinded manner by JP (FITC-albumin extravasation) and MK (serum MMP and TIMP evaluation). DM analyzed the data and wrote the manuscript, followed by a critical revision by WH, JB, DS and CW. All authors have read and approved the final manuscript.
